# Nasal paraganglioma: differential diagnosis from a radiologic and pathologic perspective

**DOI:** 10.1259/bjrcr.20160050

**Published:** 2016-11-02

**Authors:** Christine J Tolman, Olga CG Stam

**Affiliations:** ^1^Department of Radiology, Medisch Centrum Haaglanden, The Hague, Netherlands; ^2^Department of Pathology, Academisch Medisch Centrum, Amsterdam, Netherlands

## Abstract

A 33-year-old Asian male presented with spontaneous nosebleeds and olfactory sense problems for the past several years. CT scan and MRI demonstrated a large soft tissue mass in the nasal cavity and paranasal sinus with avid and homogeneous contrast enhancement, focal osseous destruction and a non-enhancing cyst at the intracranial tumour–brain margin. After complete endonasal resection, histopathological examination revealed a paraganglioma. This case highlights the non-specific imaging features of a rare paraganglioma of the anterior skull base and the differential diagnosis from both radiological and pathological perspective.

## Clinical presentation

A 33-year-old Asian male presented to the ENT clinic with complaints of spontaneous nosebleeds for the past 6 months. Furthermore, the patient also had been suffering from significant olfactory problems for more than 2 years but was reluctant to seek medical advice or any investigations for this. When asked, the patient complained of mild headaches in the anterior part of the head. He had normal vision and no hearing loss. Past medical history included kidney stones, a car accident 7 years ago and bilateral carpal tunnel syndrome, which required surgery on both sides. He had no allergies and used no medication. No tobacco, alcohol or drugs abuse. No occupational high-risk contact with carcinogens was reported. On neurological examination, he appeared a bright and oriented patient with absence of olfactory sense, intact vision, no hemianopia, normal motor function and reflexes, and normal global sensitivity. On clinical examination, a mass in the nasal cavity was suspected. Laboratory results were normal. CT and MRI demonstrated a large soft tissue mass in the nasal cavity.

## Imaging findings

**Figure 1.**
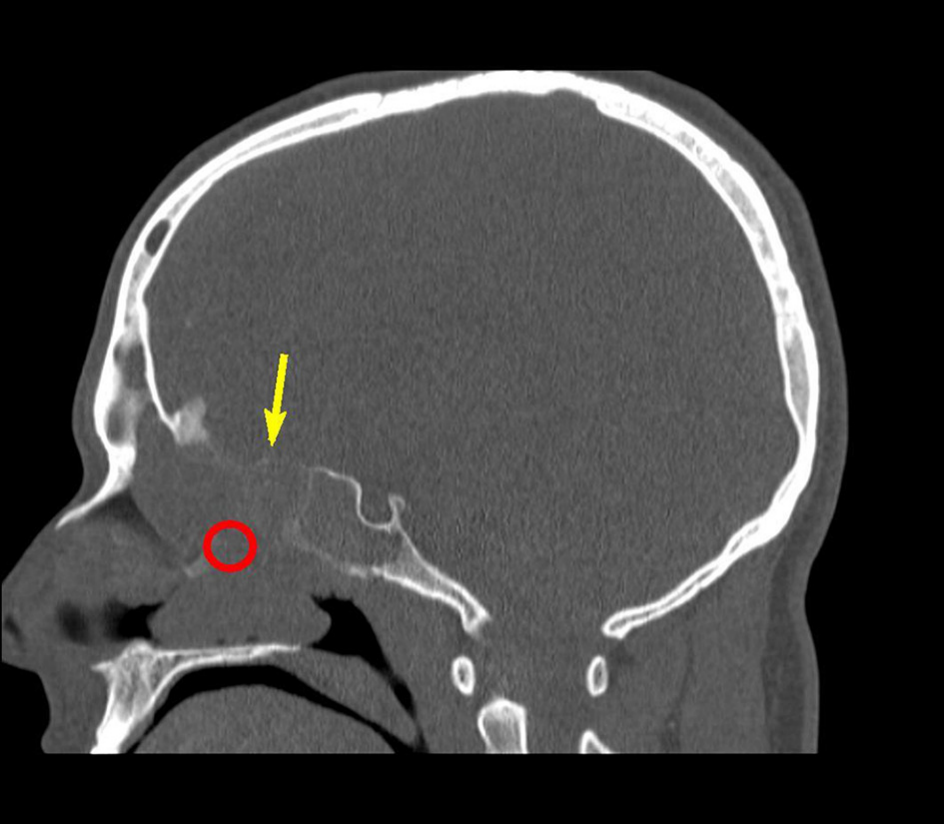
Sagittal non-enhanced CT imaging in bone window shows a soft tissue mass (red circle) and focal erosive bone changes of the orbital part of the left frontal bone (yellow arrow).

Non-enhanced CT imaging of the sinus showed a homogeneous soft tissue mass in the left nasal cavity and ethmoid with extension into the left frontal sinus. There were no focal dense areas suggestive of haemorrhage or calcifications. Slight mass effect on the left orbit and focal erosive bone changes of the orbital part of the left frontal bone, the superior orbital margin and glabella were seen (Figure 1).

**Figure 2.**
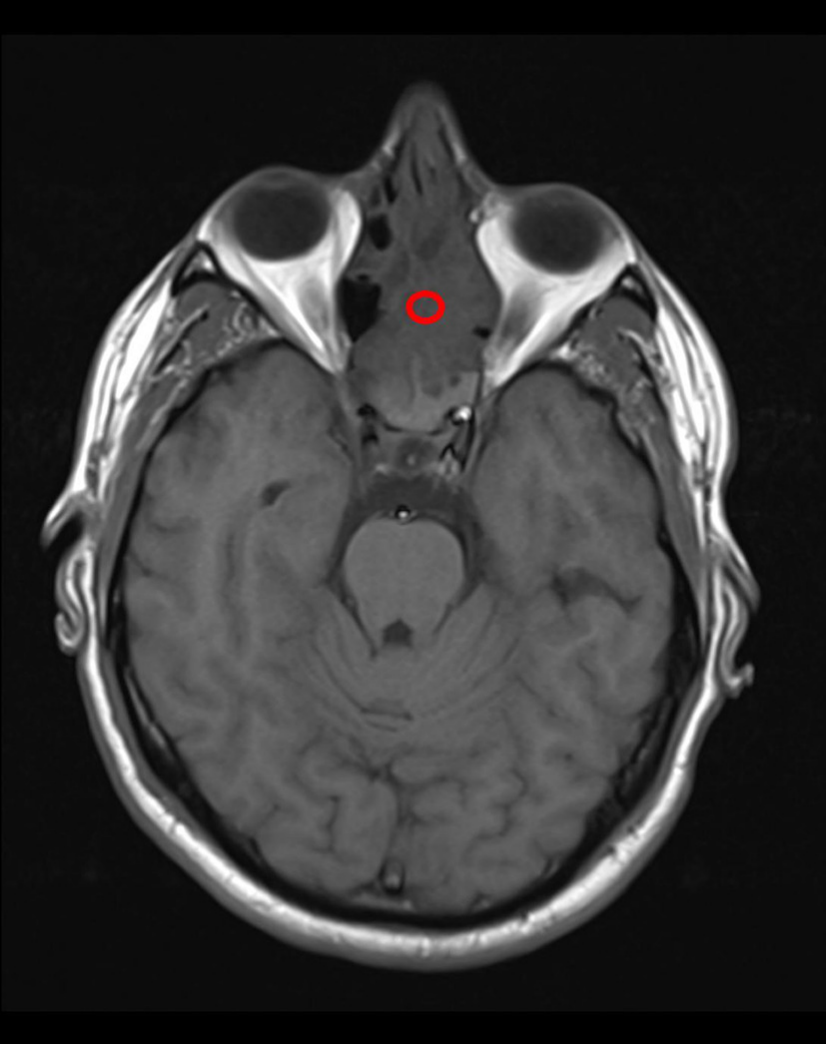
Axial *T*_1_ weighted turbo inversion recovery magnitude MRI reveals a homogeneous isointense mass in the nasal cavity (red circle).

**Figure 3.**
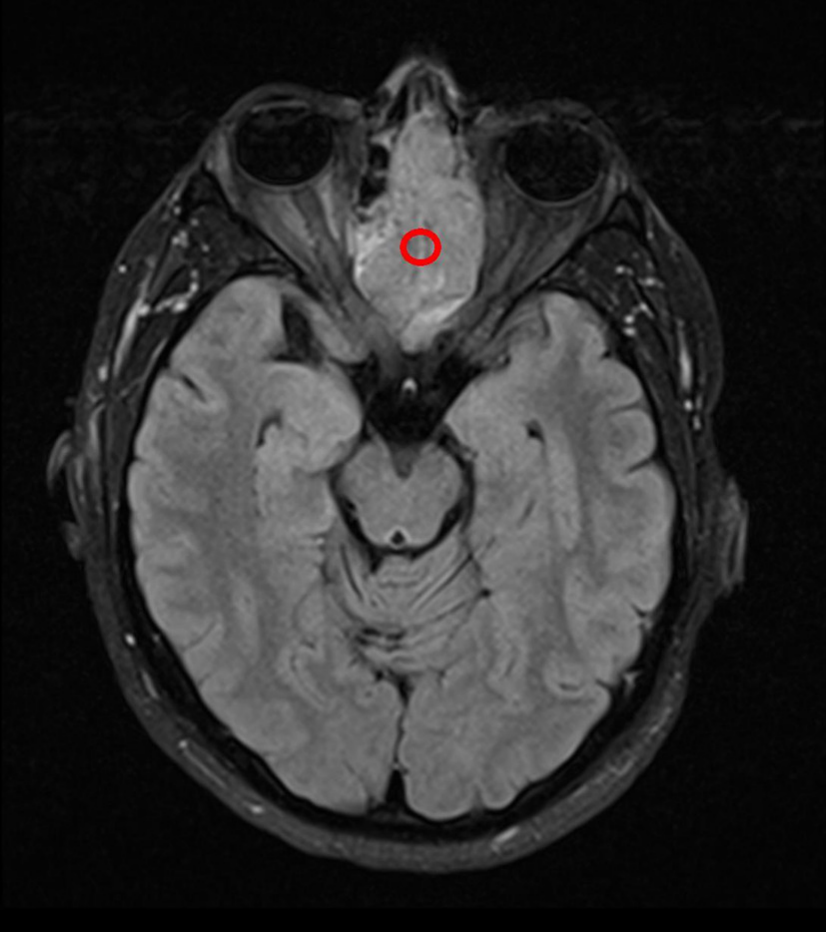
Axial *T*_2_ weighted turbo inversion recovery magnitude MRI showing a lobulated soft tissue mass (red circle) expanding the nasal septum bilaterally with slight hyperintense signal. Some hypointensities, possible flow voids, are seen.

**Figure 4.**
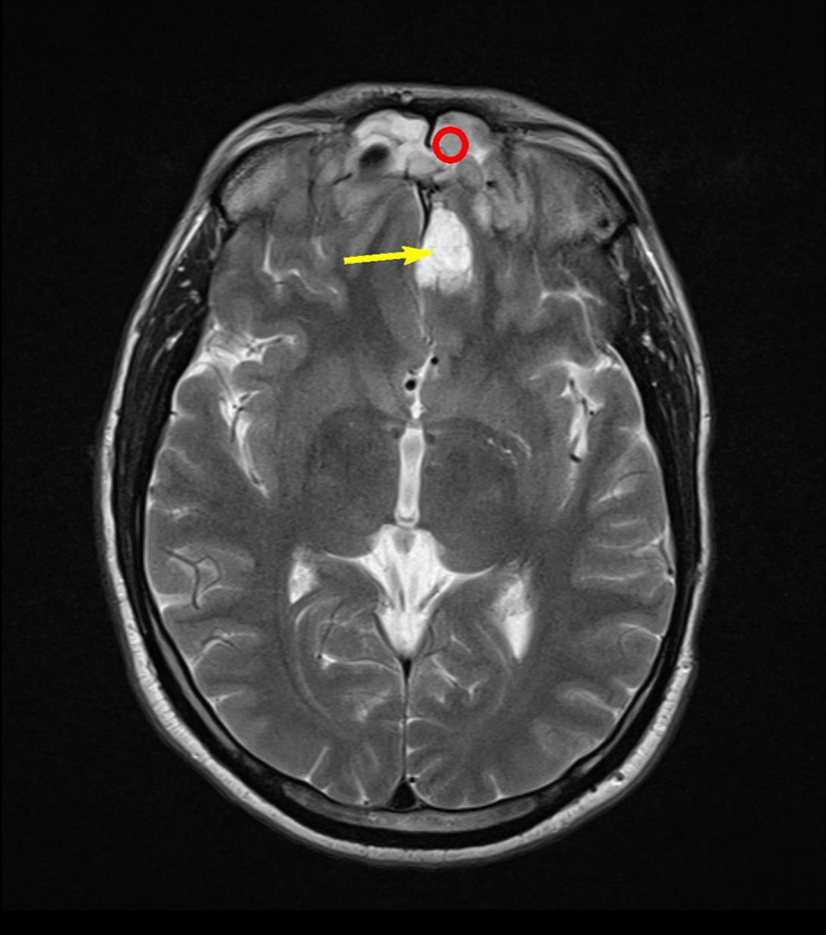
Axial *T*_2_ weighted MRI showing cranial extension of the mass (red circle) along the anterior falx into a cystic part (yellow arrow). No oedema is seen in the frontal lobe parenchyma.

**Figure 5.**
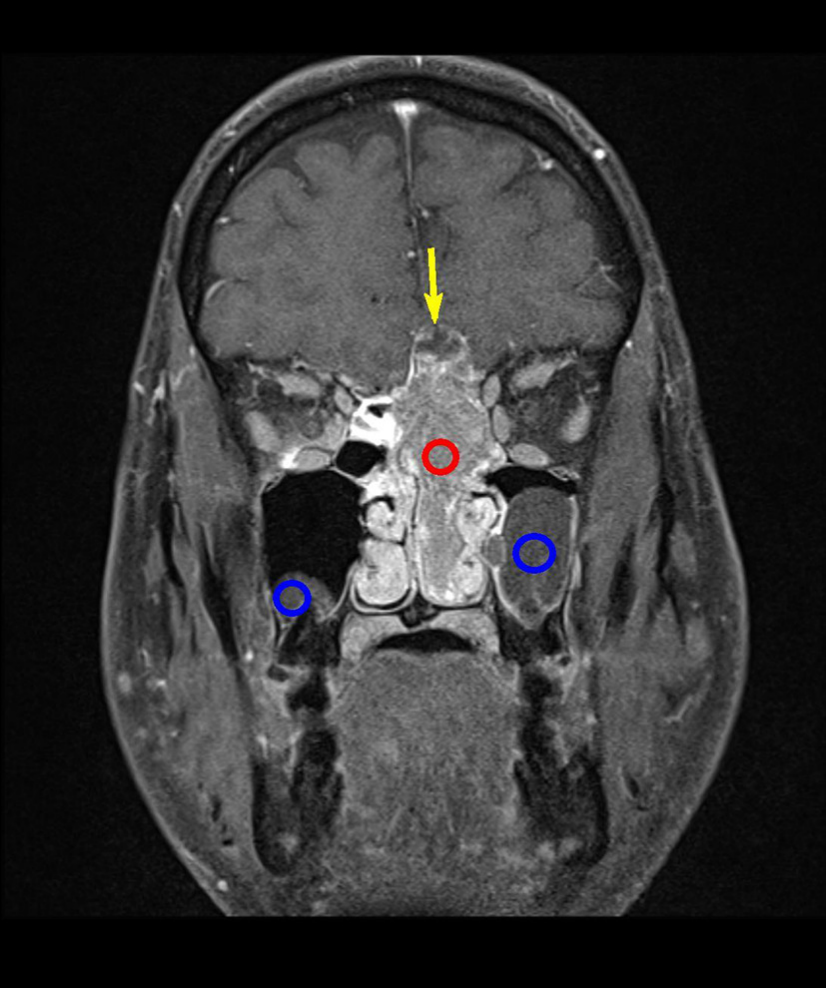
Coronal *T*_1_ weighted image with contrast and fat suppression. showing a vivid, homogeneous enhancing mass (red circle) and circumferentially enhancing border of the cranial cyst (yellow arrow). Bilateral pre-existent benign retention cysts are seen (blue circles) with no relation to the mass.

MRI demonstrated a large space-occupying, lobulated homogeneous mass sided in the left nasal cave and ethmoid. Slight extension across the midline to the right side of the nasal septum and right ethmoid cells was seen. Pre-contrast *T*_1_ signal intensity was isointense to muscle (Figure 2). On turbo inversion recovery magnitude and *T*_2_ weighted imaging, the tumour was moderately hyperintense (Figure 3), with no microcysts but some punctiform hypointensities, possible flow voids. No extension into the pterygopalatine fossa, middle cranial fossa, orbits or palate was seen. Superior extension into the left frontal sinus with cranial extension into a small cyst with high *T*_2_ signal along the left side of the anterior falx cerebri was seen. On the turbo inversion recovery magnitude series, no oedema suggestive of invasive intracranial growth was seen (Figure 4). There was intense homogeneous enhancement of the mass after contrast administration and fat suppression, and enhancement of the border of the cranial cyst (Figure 5). There was no sign of perineural growth or vascular invasion.

## Outcome

Endonasal biopsy revealed a paraganglioma. After partial embolization, radical endoscopic transnasal resection was performed. During surgery, the tumour was found to involve the bilateral nasal cavities as well as the middle nasal concha and cranial part of the septum, with growth along the lamina papyracea through the sphenoid sinus to the suprasellar space. Peroperatively, the tumour did not show an invasive pattern, but it had evidently grown through the skull base. A small cyst present in the arachnoid was attached to the left side of the tumour. Radical resection of a part of the crista galli, anterior falx and olfactory nerves was performed, followed by reconstruction with Duraform and three layers of fascia lata. Post-operative MRI showed no signs of residual tumour. The patient was maintained on bed rest for 5 days to minimize the risk of leaking liquor. During admission, there were no complications and the patient was discharged in good clinical condition. No gene mutation analysis was performed.

## Histopathology findings

**Figure 6.**
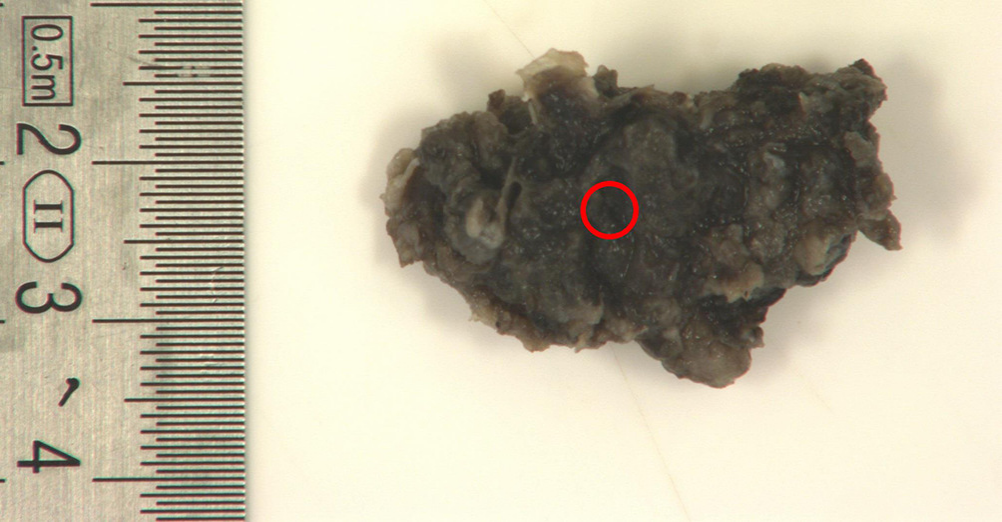
Gross photograph of anterior skull base mass. No cut sections are available. Brown/greyish soft tissue mass (red circle) is seen.

**Figure 7. Haematoxylin and eosin:**
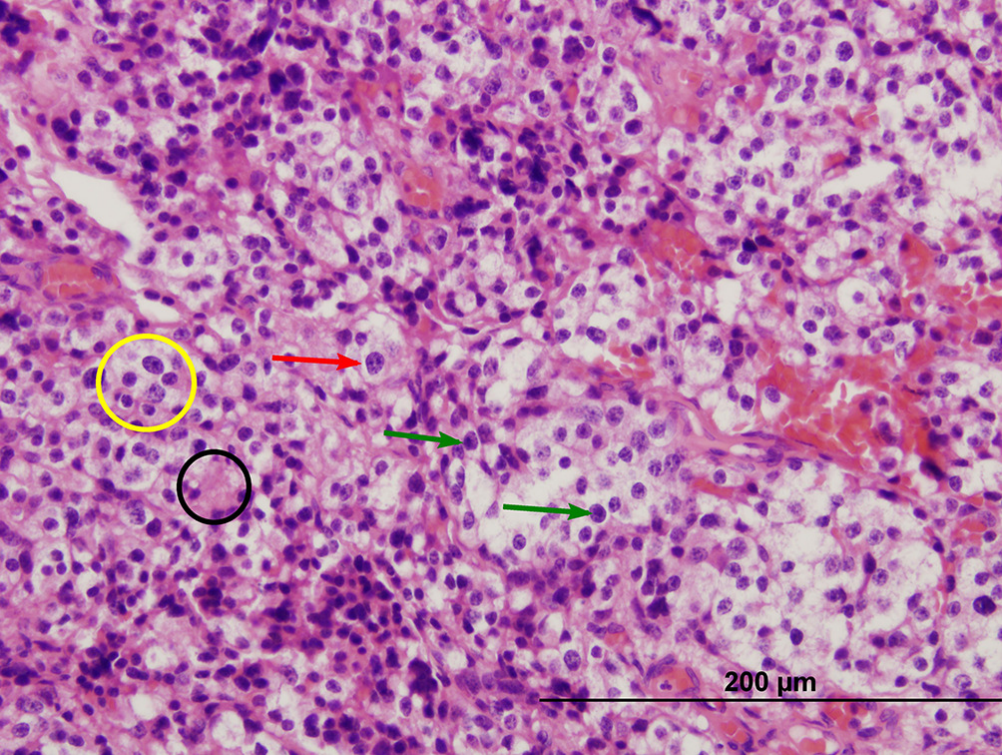
stained, 400× magnification. Bland cell proliferation with round nuclei varying in size (yellow circle) is seen. Nuclei contain clumped chromatin (red arrow) and/or prominent nucleolus (green arrows). Moderate amount of eosinophilic granular cytoplasm (black circle) is present. No (pseudo-)rosette formation or neurofibrillary background can be seen.

**Figure 8.**
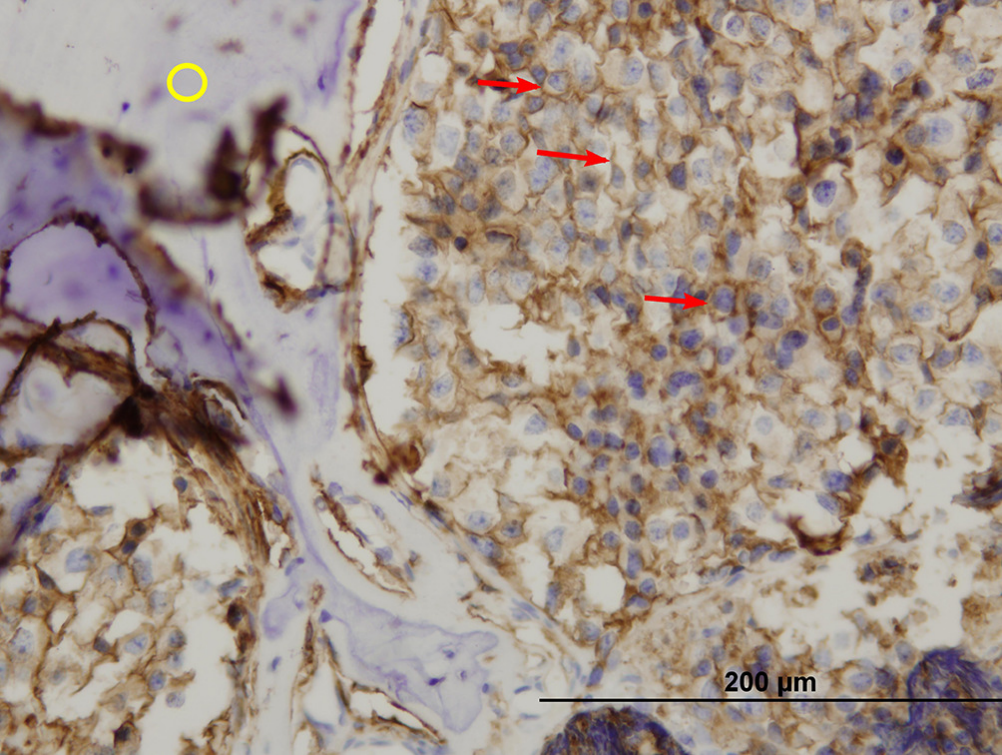
CD56 stained, 400× magnification. Brown-stained cell membrane (red arrows) and pre-existing bone (yellow circle) are seen.

**Figure 9.**
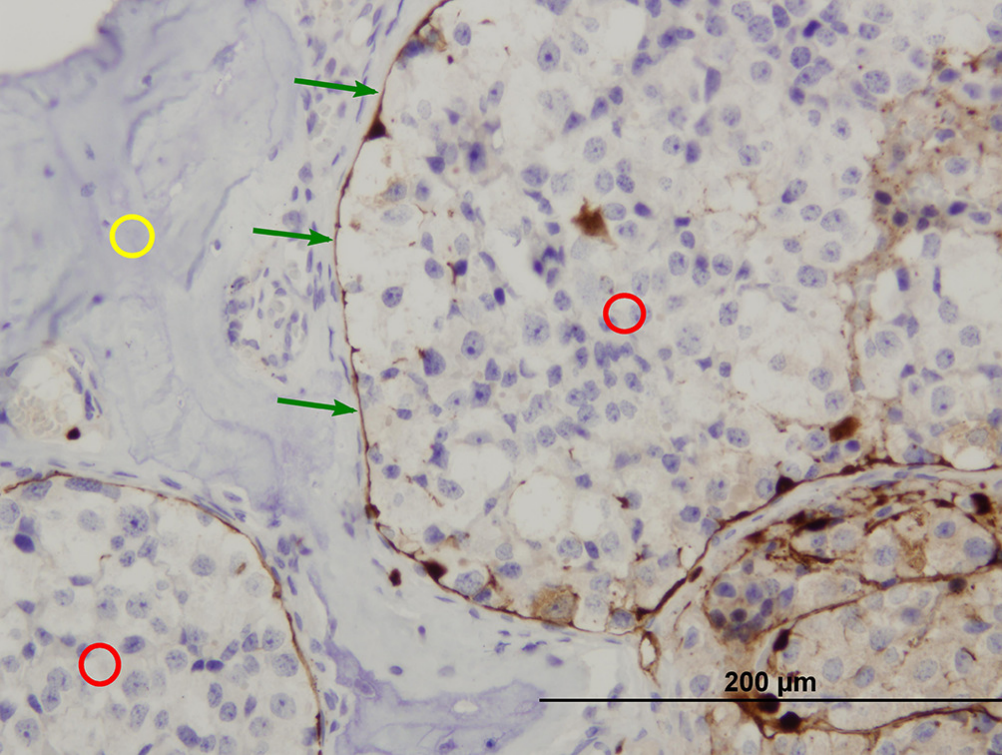
S100 stained, 400× magnification. Brown-stained sustentacular cells (green arrows) are present. There is no expression in tumour cells (red circles), with small brown artefact. Pre-existing bone (yellow circle) is seen.

**Figure 10.**
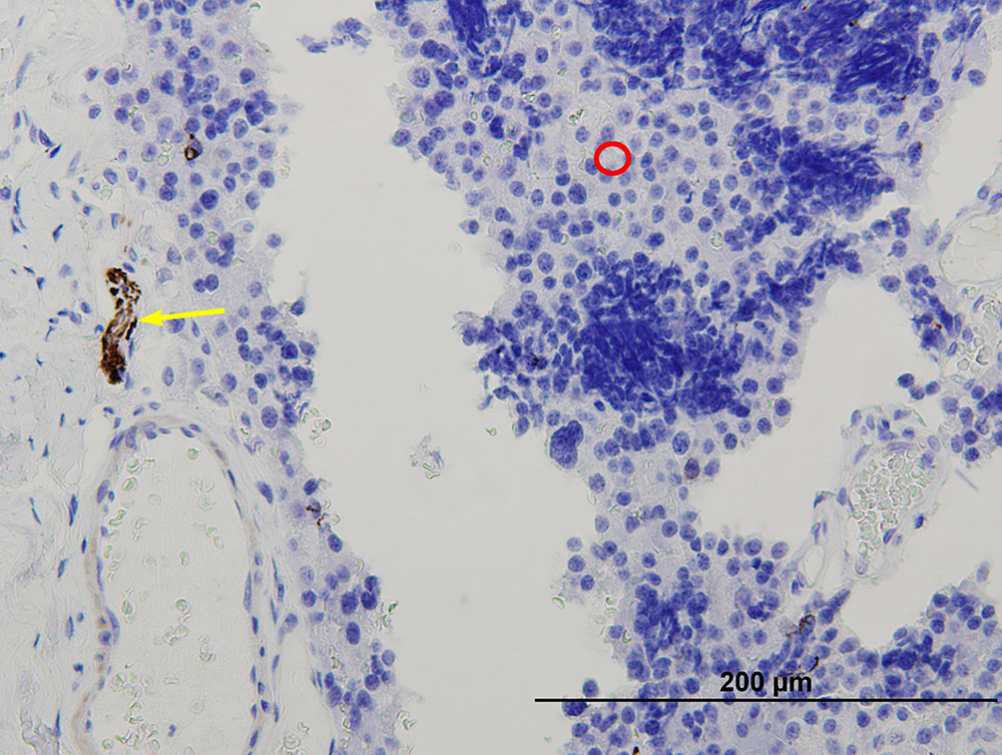
Neurofilament stained, 400× magnification. No neurofibrillary background (red circle) is seen. Brown-stained pre-existing nerve (yellow arrow) is seen.

Gross pathology demonstrated a greyish brown soft tissue mass with a small hard piece of bone on palpation (Figure 6). Microscopically, tissue fragments contained subepithelial nests and sheets of bland cells. The cells had round nuclei with nucleoli and clumped chromatin, and a moderate amount of eosinophilic granular cytoplasm. There was no (pseudo-)rosette formation or neurofibrillary background (Figure 7). Immunohistochemical examination revealed expression of CD56, chromogranin A and neuro-specific endolase in the chief cells, and expression of S100 in the sustentacular cells (Figures 8 and 9). There was no expression on neurofilament staining (Figure 10).

## Differential diagnosis on imaging

The differential diagnosis of enhancing nasal tumours with high *T*_2_ signal on imaging is broad and comprises both benign and malignant lesions.^1,2^ We will not discuss other tumour-like (congenital, traumatic or inflammatory) diagnoses and metastatic disease in this article, but focus on primary tumours of the sinonasal cavity. In this case, owing to specific imaging characteristics, the main differential diagnosis was esthesioneuroblastoma (ENB; also olfactory neuroblastoma), followed by squamous cell carcinoma (SCC) and adenocarcinoma, although our patient did not have specific high-risk factors for carcinoma (smoking).

ENB is a relatively rare, slow-growing, malignant tumour arising from the olfactory neuroepithelium and can extend into the lower anterior cranial fossa. The lesion is typically midline, centred around the cribriform plate, shows avid contrast enhancement and may variably appear heterogeneous on short tau inversion-recovery-weighted MRI.^2^ ENB may cross the midline through the nasal septum or extend into the orbits.

Both ENB and paraganglioma show homogeneous enhancement and can have a dumbbell shape, with ENB showing a typical waist at the cribriform plate. Furthermore, both show bone remodelling and erosion owing to expansive growth and finally, while ENB is characterized by its peritumoral cysts at the intracranial brain–tumour margin, paraganglioma can also have cystic parts at this location.^3^ ENB typically can have microcysts.

Of all the nasal carcinomas, SCC is the most frequent.^2^ It has a predilection for the maxillary antrum and is known for its aggressive behaviour and appearance, with heterogeneous enhancement, osseous destruction and often invasive growth into the orbits and pterygopalatine fossa, and early-stage lymph node metastases.^3^ On MRI, SCC shows intermediate *T*_1_ signal intensity and hypointense *T*_2_ signal compared with fluid. It demonstrates variable enhancement, less than the normal mucosa of the paranasal sinuses.^3^ The second most frequent is adenocarcinoma, which is more common in Asian individuals, favours ethmoid origin with nasal involvement and shows homogeneous contrast enhancement.^1^ However, both SCC and adenocarcinoma do not enhance to the same degree as ENB and paraganglioma. Poorly differentiated carcinoma cannot be excluded on MRI or CT scan, but is more common in older patients and originates mostly from the ethmoid sinus and superior nasal cavity. Imaging shows a heterogeneous soft tissue mass with aggressive features.^1^

This list of enhancing nasal tumours should be completed by mentioning sarcomas. Head and neck rhabdomyosarcoma originates most commonly in the orbits and nasopharynx and almost exclusively in children, with 70% of patients aged < 12 years, and only 12% involving the sinonasal cavities.^3^ On imaging, an inhomogeneous soft tissue mass is seen with non-specific features such as bone erosion, necrosis and early stage intracranial growth.^2^ Myeloid or granulocytic sarcoma, also called chloroma, is extremely rare and has a typically high *T*_1_ weighted imaging signal.^2^ Less likely diagnoses are melanoma, classically with high *T*_1_ and low *T*_2_ signal owing to melanin, and lymphoma, with a dense appearance on non-enhanced CT and low *T*_2_ signal owing to high cellularity. Therefore, we will also not discuss other small blue cell tumours here, such as Ewing sarcoma or primitive neuroectodermal tumour. Metastasis, plasmacytoma and malignant fibrous histiocytoma are all malignant tumours with a primary osseous origin.^1,3^

The group of benign enhancing tumours with high *T*_2_ signal in the sinonasal cavity comprises inverted papilloma; peripheral nerve sheath tumours such as schwannoma and neurofibroma (only 4% in the sinonasal tract); highly vascular lesions such as haemangioma; juvenile angiofibroma and haemangiopericytoma, with the latter having malignant potential; and benign neuroendocrine tumours such as paraganglioma.^1^

## Differential diagnosis on histopathology

On histopathological examination, the differential diagnosis is ENB, malignant melanoma and poorly differentiated carcinoma.^4^ Both poorly differentiated carcinomas and melanomas are negative for neuroendocrine markers. Poorly differentiated carcinomas of the sinonasal tract are more aggressive, and thus show (higher) mitotic activity, occasional necrosis, marked nuclear pleomorphism and an infiltrative growth pattern. Melanomas are diffusely positive for S100.^5^ The chief cells of both ENB and paraganglioma show expression for neuroendocrine markers (synaptophysin, chromogranin-A and neuro-specific endolase in the cytoplasm, and CD56 on the cell membrane) and a lining of flattened S100-positive sustentacular cells.^4,5^

However, paragangliomas can be distinguished by the bland appearance of their uniform polygonal chief cells arranged in well-demarcated nests (Zellballen) and sheets, and a background of eosinophilic granular cytoplasm. The chief cells contain round nuclei that vary in size with clumped chromatin or a prominent nucleolus, and a moderate-to-abundant amount of eosinophilic granular cytoplasm. Paragangliomas are not mitotically active, whereas ENB has a high k67 index rate.^4,5^ On the other hand, ENB is known for (pseudo-)rosette formation and moreover, its abundant neurofibrillary background for which a neurofilament immunostain can be used. Its nuclear characteristics are fine chromatin and no or inconspicuous nucleoli.^4^

## Discussion

Paragangliomas (synonyms: glomus tumour, chemodectoma, perithelioma, fibroangioma and sympathetic nevi) are tumours of the paraganglia, defined as chains of tissue originating from the extra-adrenal neuroendocrine system. According to the World Health Organization, they account for less than 2% of all soft tissue tumours^2^ and 0.6% of all tumours in the head and neck region, with the four most common locations of occurrence being the carotid bifurcation, the jugular foramen, the vagus nerve and the middle ear, also called glomus caroticum, glomus jugulare, glomus vagale, and glomus tympanicum or glomus jugulotympanicum, respectively.^6^ Among other locations are the larynx, thyroid, nasopharynx, orbit, tongue and the paranasal sinus.

Paragangliomas of the sinonasal tract are extremely rare, with less than 40 case reports described.^7,8^ The exact origin is mostly the ethmoid sinus, followed by the lateral nasal wall and middle turbinate.^6^ Because of its slow growing and expansive behaviour, patients present at an older age and late in the course of the disease with nasal obstruction, epistaxis, facial swelling, pain and loss of olfaction. Most patients are between 40 and 60 years old, with a slight female predilection.^6^ Cases of paediatric paragangliomas suggest a genetic predisposition. Currently, we discriminate between isolated paragangliomas (75%) and hereditary paragangliomas (25%) which are often multiple, for instance, in multiple endocrine neoplasia syndrome type 2A and 2B. The latter more often occur bilaterally (10%). Screening for multiple germline mutations related to familiar head and neck paragangliomas can be performed, of which succinate dehydrogenase complex subunits B and D are the most important for predicting a malignant nature.^9,10^ While all paragangliomas contain neurosecretory granules, only in 1–3% of cases is the secretion of hormones such as catecholamines abundant enough to cause symptoms in the same way as pheochromocytomas (intramedullary paragangliomas), such as by hypertension and can lead to pertinent laboratory abnormalities.^10^

Paragangliomas are vascular, expansive and sometimes destructive tumours. They have a lobulated and sometimes dumbbell-shaped appearance, show a slightly hyperintense signal on *T*_2_ sequence, and avid and homogeneous contrast enhancement on both CT scan and MRI. The classical paraganglioma has a “salt and pepper” appearance on both *T*_1_ and *T*_2_ weighted images, which is of high diagnostic value when seen; the salt-like areas are high signal areas secondary to subacute haemorrhage, whereas the pepper-like areas are low signal intensity flow voids or vascular channels.^6–8^ A similar appearance may be seen on *T*_2_ sequences or post-contrast *T*_1_ sequences in patients with juvenile nasopharyngeal angiofibroma.^3^ Owing to slow growth, adjacent bone erosion and remodelling occur frequently.^3,6–8^ Larger masses can have degenerated cystic parts, not to be mistaken for necrosis. On angiography, a prolonged tumour blush is seen owing to hypervascularity. Indium-111 octreotide is used for nuclear imaging of paragangliomas.^6^

When there is clinical suspicion of a mass in the paranasal sinus, diagnostic work-up will routinely include at least a CT scan and MRI of the head and neck to determine the skull base and intracranial extent and unexpected multiple/bilateral paragangliomas or metastases. Thoracic and abdominal CT scans are performed on indication, also dependent on genetic mutation analysis and risk factors.

CT scan and MRI complement each other; CT scan of the sinus and/or brain is superior to MRI for bone evaluation; benign, expansive, slow-growing tumours that cause bone remodelling and erosion can be differentiated from lytic, aggressive, malignant behaving tumours.^3^ CT imaging can also show calcifications.^1,2^ We suggest non-enhanced CT scan, with optional contrast-enhanced series.

MRI excels in demonstrating soft tissues and features such as high or low cellularity, central necrosis or (micro-)cysts, and vascular channels or haemorrhage. Moreover, MRI displays the exact location, size and possible extension into the pterygopalatine fossa, middle cranial fossa, orbits and brain. These features have an impact on diagnostic considerations and consequent surgical or therapeutic planning.^3^ We suggest *T*_1_ weighted MRI with and/or without fat suppression, *T*_2_ and *T*_1_ weighted image with fat suppression and contrast administration, preferably in three-dimensional reconstructions, and optional susceptibility weighted imaging for (micro-)haemorrhage. Diffusion-weighted imaging and dynamic contrast-enhanced MRI may be of additional value.^11^

Oncologic guidelines and treatment algorithms of head and neck paragangliomas state that pre-operative fine-needle aspiration biopsy is optional. This can be performed safely by the ENT surgeon to establish a diagnosis. If surgery is contemplated, angiography is indicated to evaluate tumour blood supply and bilateral cerebral blood supply and possible vessel invasion.^12^

## Treatment

Resection is the treatment of choice, often performed by both ENT surgeons and neurosurgeons with an endoscopic endonasal approach. The objective is to obtain disease-free resection margins, which is challenging since paragangliomas grow around normal vital structures. There is a preference for pre-operative embolization in tumours larger than 3 cm in order to reduce the vascularity and size of the tumour, leading to a smaller amount of peroperative blood loss.^13^ Some authors describe the usefulness of radiotherapy. Chemotherapy thus far plays no role in the therapy of paragangliomas.^8,10,12^

## Prognosis

The majority (97%) of paranasal paragangliomas are benign.^6–8,13^ Prognosis is partly related to the location of origin; the skull base region has therefore a less favourable outcome since radical resection is more difficult to perform.^6^ Chances for malignant paraganglioma increase with a mutation in the succinate dehydrogenase (*SDHB*) gene.^9^ When malignant, sinonasal paragangliomas show aggressive behaviour with rapid local growth, vascular invasion and metastases to brain, lung, skin, bone and regional lymph nodes,^8,10^ especially in recurrent tumours. Recurrence rate in the head and neck region is about 10% and malignant transformation in a recurrent tumour is not infrequent (2–13%);^8^ cases have been described of malignant recurrence after 13 years of follow-up.^10^ Hereditary paragangliomas are more often bilateral (10%) and multiple, so screening and follow-up of other locations in these patients is also necessary. Thus far, no consensus has been reached about the duration of follow-up and the imaging of choice. Nuclear imaging might play an important role in the search for local recurrence and (lymph node) metastases.^10^

## Conclusions

The broad radiological differential diagnosis of enhancing (para-)nasal tumours makes it difficult to diagnose the extremely rare phenomenon of benign paraganglioma of anterior skull base, which shares most features with malignant ENB. Histopathology and immunohistochemistry lead to the final diagnosis and appropriate treatment.

## Learning points

1. Benign tumours can behave aggressively on imaging.
2. Imaging features of enhancing tumours of the sinonasal tract are often non-specific. There is a broad radiological differential diagnosis of both benign and malignant tumours.
3. Histopathology plays a key role in the final diagnosis.

## Acknowledgment

Special appreciation goes to the faculty of Pathology of the Academic Centre of Amsterdam for technical, scientific and mental support for the admission of this case to the American Institute for Radiologic Pathology and publication of this manuscript.

## Consent

Informed consent to publish images and data was obtained and is held on record.
